# Network Analysis of Outpatients to Identify Predictive Symptoms and Combinations of Symptoms Associated With Positive/Negative SARS-CoV-2 Nasopharyngeal Swabs

**DOI:** 10.3389/fmed.2021.685124

**Published:** 2021-07-20

**Authors:** Hervé Spechbach, Frédérique Jacquerioz, Virginie Prendki, Laurent Kaiser, Mikaela Smit, Alexandra Calmy, François Chappuis, Idris Guessous, Julien Salamun, Stéphanie Baggio

**Affiliations:** ^1^Division of Primary Care Medicine, Geneva University Hospitals, Geneva, Switzerland; ^2^Division of Tropical and Humanitarian Medicine, Geneva University Hospitals, Geneva, Switzerland; ^3^Geneva Center for Emerging Viral Diseases, Geneva University Hospitals, Geneva, Switzerland; ^4^Division of Infectious Diseases, Geneva University Hospitals, Geneva, Switzerland; ^5^Division of Internal Medicine for the Aged, Geneva University Hospitals, Geneva, Switzerland; ^6^Laboratory of Virology, Geneva University Hospitals, Geneva, Switzerland; ^7^Faculty of Medicine, University of Geneva, Geneva, Switzerland; ^8^HIV/AIDS Unit, Department of Infectious Diseases, Geneva University Hospitals, Geneva, Switzerland; ^9^Division of Prison Health, Geneva University Hospitals, Geneva, Switzerland; ^10^Office of Corrections, Department of Justice and Home Affairs of the Canton of Zurich, Zurich, Switzerland

**Keywords:** SARS-CoV-2, COVID-19, outpatient, diagnosis, predictive symptoms, clinical features, combination of symptoms

## Abstract

**Background:** Limited data exist on early predictive clinical symptoms or combinations of symptoms that could be included in the case definition of coronavirus disease 2019 (COVID-19), particularly for mild-to-moderate disease in an outpatient setting.

**Methods:** A cohort study of individuals presenting with clinical symptoms to one of the largest dedicated networks of COVID-19 test centers in Geneva, Switzerland, between March 2 and April 23, 2020. Individuals completed a symptom questionnaire, received a nurse-led check-up, and nasopharyngeal swabs were obtained. An analysis of clinical features predicting the positivity and negativity of the SARS-CoV-2 RT-PCR test was performed to determine the relationship between symptoms and their combinations.

**Results:** Of 3,248 patients included (mean age, 42.2 years; 1,504 [46.3%] male), 713 (22%) had a positive RT-PCR; 1,351 (41.6%) consulted within 3 days of symptom onset. The strongest predictor of a positive SARS-CoV-2 RT-PCR was anosmia, particularly in early disease, followed by fever, myalgia, and cough. Symptoms predictive of a negative test were breathing difficulties, abdominal symptoms, thoracic pain and runny nose. Three distinct networks of symptoms were identified, but did not occur together: respiratory symptoms; systemic symptoms related to fever; and other systemic symptoms related to anosmia.

**Conclusions:** Symptoms and networks of symptoms associated with a positive/negative SARS-CoV-2 RT-PCR are emerging and may help to guide targeted testing. Identification of early COVID-19-related symptoms alone or in combination can contribute to establish a clinical case definition and provide a basis for clinicians and public health authorities to distinguish it from other respiratory viruses early in the course of the disease, particularly in the outpatient setting.

## Introduction

On January 12, 2020, several severe pneumonia cases were identified in Hubei Province, China, and later related to coronavirus disease 2019 (COVID-19) caused by the novel severe acute respiratory syndrome coronavirus 2 (SARS-CoV-2) (1). On March 11, 2020, the World Health Organization (WHO) declared the outbreak a pandemic (2). At present, the number of confirmed cases exceeds 100 million worldwide, with more than 2.2 million deaths (3).

The clinical manifestations of COVID-19 comprise a wide range of symptoms (4, 5), with most infected individuals experiencing only mild or subclinical illness, particularly in the early phase. To date, the focus has primarily been on describing clinical symptoms among hospitalized patients, which mainly include respiratory symptoms (e.g., cough, shortness of breath) and fever associated with myalgia and fatigue, similar to early case reports from China (6–9). The set of symptoms outlined by WHO for the COVID-19 case definition (10) has changed over time to reflect the spectrum of reported symptoms, including non-respiratory manifestations, such as loss of smell/taste (11), neurological symptoms (12), ocular manifestations (13), and dermatological signs (14). Some studies have also described asymptomatic presentations (15) or the presence of milder respiratory symptoms (4).

Studies analyzing the early clinical symptoms as risk factors for testing positive for SARS-CoV-2 in an outpatient setting are sparse (16) and often focus on specific populations, such as veterans (17) or healthcare workers (18), rather than the general population. In the absence of proven prophylactic candidates and at the beginning of widespread vaccination on an unprecedented scale, the containment of SARS-CoV-2 currently focuses on the interruption of transmission, notably through rapid and targeted testing, prompt isolation and contact tracing. However, the clinical definition of COVID-19 remains to be fully established, particularly among paucisymptomatic patients (19, 20). Importantly, this would facilitate targeted testing at the early stage of the disease and be particularly valuable in resource-limited settings or in a situation of SARS-CoV-2 high incidence where costly laboratory tests are restricted.

Efforts have been made to identify more specific symptoms or combinations of symptoms to discriminate between COVID-19 and other respiratory tract infections. Some authors showed that COVID-19 infection had a similar onset to other types of pneumonia (21), whereas, others reported that it initiates with fever, as for other coronavirus-related diseases, but unlike influenza that starts with cough (22). New approaches, including network analysis, a graph, theory-based methodology, have allowed to visualize and identify the relationship between several symptoms and their combinations associated with a disease. For example, network analysis has been used in oncology (23) and for neuropsychiatric symptoms associated with COVID-19 since the beginning of the pandemic by establishing three symptom categories (24).

In the present study, we aimed to identify early clinical symptoms predictive of a positive or negative SARS CoV-2 test in nasopharyngeal swab material based on reverse-transcription polymerase chain reaction (RT-PCR) among a large cohort of outpatients presenting with mild-to-moderate symptoms. We also established how symptoms combine together to form three distinct categories.

## Methods

### Study Design and Setting

The study was approved by the regional ethics committee (no. 2020-00813).

Geneva University Hospitals (HUG) is the largest of the five university hospitals in Switzerland and one of the largest hospitals in Europe. It serves the region of Geneva and its surrounding population (approximately 800,000 persons) and operates across several sites, including eight hospitals, two clinics and 30 outpatient care centers. We included all individuals aged 16 years old and over consulting for a suspicion of SARS-CoV-2 infection at any of the dedicated outpatient test centers within the HUG network. Patients who refused the use of their personal data for research purposes were excluded.

At the beginning of March 2020, four dedicated test centers were set up within the existing HUG outpatient facilities. In each center, a standardized triage oriented patients with severe symptoms to the emergency room, while other patients were evaluated and tested on site. Patients were screened according to the Swiss Federal Office of Public Health definition of suspected cases based on WHO recommendations. Data for all eligible patients presenting to the centers were collected from March 2, 2020, to April 23, 2020, during the first wave of the pandemic. Data collected during the week of March 18, 2020, to March 26, 2020, were excluded as only patients with fever and cough were tested due to a lack of logistic testing possibilities.

### Variables and Data Sources

All outpatients completed a questionnaire upon admission with the support of a nurse, a trained medical student or a physician. The questionnaire collated structured information on sociodemographic and medical factors including age, gender, social determinants, clinical symptoms, and onset, comorbidities, and risk factors for more severe illness. The Treatment section was unstructured and completed by the patients in the free-text comments. Vital signs were only noted if the patient presented comorbidities. In the present analysis, we focused on clinical symptoms. Our questionnaire was adapted throughout the study period in accordance with best evidence on the evolution of the disease and its symptoms. Specifically, symptoms initially included runny nose, sore throat, cough (dry and with sputum), fever, muscle pain, and chills. Anosmia and headache were added on March 24, 2020. Breathing difficulties, abdominal symptoms, fatigue and thoracic pain were added on April 1, 2020. The above-mentioned additional symptoms were reported as free text in the section “other symptoms” in early versions of the questionnaire and were subsequently recoded. Participants who did not report any symptoms were considered asymptomatic. Risk factors for severe illness or complications included hypertension, chronic respiratory diseases, diabetes, heart diseases, and immunosuppression.

### Outcome Variable: RT-PCR SARS-CoV-2

The main outcome was a positive SARS-CoV-2 RT-PCR test performed on a nasopharyngeal swab. All swabs were processed at the HUG virology laboratory, which is also the Swiss national reference laboratory for SARS-CoV-2. RT-PCR was performed according to manufacturers' instructions on various platforms, including initially an in-house method using eMAG (bioMérieux, Marcy l'Etoile, France) and the Charité/Berlin RT-PCR protocol, followed by the BD SARS-CoV-2 reagent kit for BD Max system (Becton Dickinson & Co, Franklin Lakes, NJ, USA) and Cobas 6800 SARS CoV2 RT-PCR (Roche Diagnostics, Rotkreuz, Switzerland).

### Statistical Analyses

We computed descriptive statistics for demographics using percentages, means, and standard deviations, according to the distribution of variables. We then computed the prevalence rate of infected cases, together with 95% confidence intervals (CI). Third, we computed descriptive statistics (percentages) for symptoms in the whole sample and then among participants infected, not infected, and not tested. Predictive clinical symptoms for positive or negative SARS-CoV-2 RT-PCR tests were evaluated using multiple logistic regression, which included all symptoms as potential predictors of a SARS-CoV-2 positive test. A clinical symptom was considered to be predictive if it had a *p*-value <0.05.

To investigate further COVID-19 symptomatology, we then used network analysis, a recent data-driven method that tests the direct relationships of a constellation of symptoms and identifies how symptoms relate to each other and how they combine together (25, 26). Networks were estimated using the IsingFit model designed for binary variables (27) and a penalty parameter to shrink small coefficients to zero using the graphical LASSO algorithm (28). We tested whether symptoms were combined together using the Spinglass algorithm, a modularity-community detection algorithm (29). Usual model accuracy checks were performed, including edge-weight accuracy and centrality stability (30).

Finally, four sensitivity analyses were conducted. As analyses were performed at the visit level, meaning that we did not consider whether visits were nested within participants, we first used mixed-effect logistic regression to test whether results were similar when taking into account the network of symptoms. As only a small subset (2.5%) of participants came more than once to the test center, the main results were presented at the visit level. Second, results of the logistic regressions were controlled for age and gender. We also performed a logistic regression controlled for the number of risk factors. Fourth, an analysis limited to a sub-sample of outpatients who completed the last version of the questionnaire was performed, which included all symptoms (no recoding needed). Findings were similar to those already observed in the previous analyses. Descriptive statistics and logistic regressions were performed using Stata 15. The network analysis was computed using R 3.6.2 and the packages IsingFit 0.3.1 (network estimation and visualization, using default parameters), igraph 1.2.5 (community detection analysis) and bootnet 1.3 (model accuracy).

## Results

### Patient Demographics and Clinical Characteristics

Among the 6,018 visits to the test centers during the study period, 2,744 (45.6%) were excluded as they corresponded to the widespread test strategy targeting asymptomatic healthcare workers. Of the remaining 3,274 visits, 20 (0.6%) were excluded as patients did not consent to the use of their data; six additional patients were excluded as they were probable SARS-CoV-2 cases with a non-detected RT-PCR. A total of 3,248 visits were included in the final analysis. Among these, 3,166 (97.5%) individuals came only once, 81 (2.5%) came twice, and one participant came three times (mean [SD] age 42.2 [14.8] years; 46.3% male). Detailed demographics are reported in Table 1. Evaluation of symptoms among patients presenting for testing are shown in Table 2. Most presented with cough (65.9%), runny nose (45.6%) or muscle pain (45.6%) and visits generally occurred in the first 3 days after symptom onset (Table 2).

**Table 1:** Demographics and sample characteristics.

	**Overall**	**SARS-CoV-2 test**		
		**Positive**	**Negative**	**No test**
	***n*** **= 3,248**	***n*** **= 712**	***n*** **= 2,467**	***n*** **= 69**
**No. of patients (%, ***n***)**				
1 visit	97.5 (3,166)	97.8 (696)	97.4 (2,402)	98.6 (68)
2 visits	2.5 (81)	2.2 (16)	2.6 (64)	1.4 (1)
3 visits	0.0 (1)	-	0.0 (1)	-
**Age (mean, SD)**				
	42.17 (14.78)	42.71 (14.00)	42.11 (14.93)	38.88 (17.19)
**Gender (%, ***n***)**				
Female	53.7 (1,744)	49.7 (354)	55.0 (1,358)	46.4 (32)
Male	46.3 (1,504)	50.3 (358)	45.0 (1,109)	53.6 (37)
**Risk factors (%, ***n***)**				
Hypertension	10.9 (353)	10.3 (73)	11.3 (278)	3.0 (2)
Chronic respiratory diseases	13.2 (427)	10.1 (72)	14.2 (349)	8.7 (6)
Diabetes	4.6 (149)	4.9 (35)	4.5 (112)	2.9 (2)
Heart diseases	4.7 (152)	3.8 (27)	5.0 (123)	3.0 (2)
Immunosuppression	6.5 (210)	4.9 (35)	7.0 (173)	2.9 (2)
No. of risk factors (mean, SD)	0.49 (0.72)	0.34 (0.70)	0.42 (0.73)	0.21 (0.48)

*SD, standard deviation*.

**Table 2:** Description of symptoms.

	**Overall**	**SARS-CoV-2 test**		
		**Positive**	**Negative**	**No test**
**Number of patients**				
	*n* = 3,248	*n* = 712	*n* = 2,467	*n* = 69
**Symptoms (%, ***n***)**				
Runny nose	45.6 (1,481)	43.0 (306)	46.9 (1,156)	27.5 (19)
Sore throat	41.8 (1,356)	34.7 (247)	44.3 (1,093)	23.2 (16)
Muscle pain	45.6 (1,481)	58.4 (416)	42.6 (1,050)	21.7 (15)
Chills	34.2 (1,111)	40.3 (287)	32.9 (812)	17.4 (12)
Fever	32.8 (1,066)	47.6 (339)	28.9 (713)	20.3 (14)
Headache	44.8 (1,456)	50.0 (356)	43.9 (1,084)	23.2 (16)
Anosmia	13.7 (444)	31.7 (226)	8.8 (217)	1.5 (1)
Abdominal symptoms	23.2 (751)	20.1 (143)	24.3 (600)	11.6 (8)
Fatigue	31.7 1,030	28.8 (205)	33.1 (817)	11.6 (8)
Cough	65.9 (2,139)	71.1 (506)	65.2 (1,609)	34.8 (24)
Difficulty breathing	23.9 (777)	16.7 (119)	26.5 (653)	7.3 (5)
Thoracic pain	17.6 (571)	12.9 (92)	19.1 (472)	10.1 (7)
No symptom	3.0 (96)	1.0 (7)	2.9 (71)	26.1 (71)
Total no. of symptoms (mean, SD)	4.35 (2.25)	4.67 (2.13)	4.32 (2.26)	2.17 (1.95)
**Time since symptom onset (%, ***n***)**				
0–3 days	41.6 (1,351)	41.7 (297)	41.7 1,029	36.2 (25)
4–7 days	26.5 (862)	31.6 (225)	25.3 (625)	17.4 (12)
8 days or more	24.1 (783)	22.5 (160)	24.9 (613)	14.5 (10)
Missing^a^	7.8 (252)	4.2 (30)	8.1 (200)	31.9 (22)

a *Including asymptomatic visits (n = 88)*. *SD, standard deviation. Combination of symptoms highlighted by color: light gray = systemic 1 (“flu-like” syndrome); orange = respiratory symptoms; and white = systemic 2. Dark-gray symptoms were not included in any community*.

### Infection Rate

Among the 3,248 tests performed, 712 (21.9%) were positive (95% CI, 20.5–23.4). Of these, 98.3% (*n* = 707) had a first positive test result and 0.7% (*n* = 5) had a second positive test (individuals previously diagnosed positive). A total of 2467 (76.0%; 95% CI, 74.5–77.4) patients tested negative; 69 (2.1%; 95% CI, 1.7–2.7) were not tested as they did not present with symptoms that made them eligible according to national recommendations at that time.

### Symptoms Associated With SARS-CoV-2 Infection

The multivariate analysis suggested that predictive symptoms for a positive SARS-CoV-2 test were anosmia (odds ratio [OR], 5.85 [95% CI, 4.62–7.40]; *p* < 0.001), followed by fever (OR, 1.96 [95% CI, 1.62–2.37]; *p* < 0.001), muscle pain (OR, 1.71 [95% CI, 1.41–2.07]; *p* < 0.001), and cough (OR, 1.53 [95% CI, 1.25–1.88]; *p* < 0.001). Symptoms predicting a negative SARS-CoV-2 test were difficulty in breathing (OR, 0.53 [95% CI, 0.41–0.68]; *p* < 0.001), sore throat (OR, 0.63 [95% CI, 0.52–0.76]; *p* < 0.001), abdominal symptoms (OR, 0.69 [95% CI, 0.54–0.88]; *p* < 0.002), thoracic pain (OR, 0.70 [95% CI, 0.53–0.92]; *p* < 0.012), and runny nose (OR, 0.76 [95% CI, 0.63–0.92]; *p* < 0.004) (Table 3).

**Table 3:** Multiple logistic regression with all symptoms predicting the SARS-CoV-2 RT-PCR result (*n* = 3,179).

**Symptoms**	**Odds ratio**	***p*****-value**
Runny nose	0.76 (0.63; 0.92)	0.004
Sore throat	0.63 (0.52; 0.76)	<0.001
Muscle pain	1.71 (1.41; 2.07)	<0.001
Chills	0.98 (0.80; 1.19)	0.832
Cough	1.53 (1.25; 1.88)	<0.001
Fever	1.96 (1.62; 2.37)	<0.001
Headache	1.12 (0.92; 1.37)	0.245
Anosmia	5.85 (4.62; 7.40)	<0.001
Abdominal symptoms	0.69 (0.54; 0.88)	0.002
Fatigue	0.83 (0.66; 1.03)	0.089
Difficulty breathing	0.53 (0.41; 0.68)	<0.001
Thoracic pain	0.70 (0.53; 0.92)	0.012

**Figure 1:**
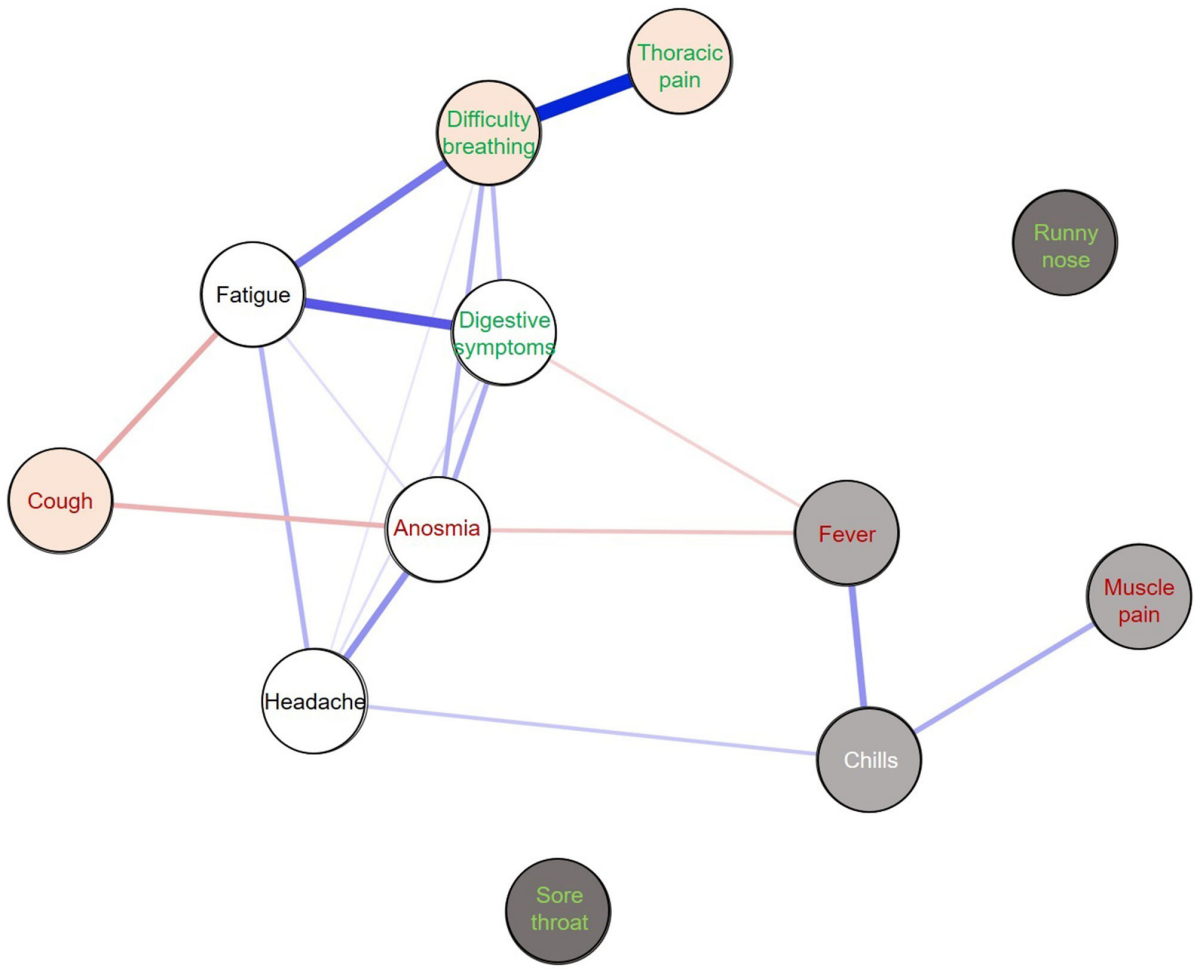
Network of symptoms among positive SARS-CoV-2 RT-PCR tests (*n* = 707). The different colored circles correspond to distinct communities: light gray = systemic 1 (“flu-like” syndrome); orange = respiratory symptoms; white = systemic 2. Dark-gray symptoms were not included in any community. Predictive symptoms (multivariate analysis) for a positive RT-PCR test are shown in red and predictive symptoms for a negative RT-PCR test are shown in green. Blue edges correspond to positive links between symptoms and red edges to negative links. The thicker the line, the stronger the link.

Figure 1 presents the results of the network analysis of symptoms associated with a positive SARS-Cov-2 test. Symptoms were found to gather into three distinct symptom combinations with one or more symptoms predictive for a positive SARS-CoV-2 test within each combination: systemic symptoms including chills, fever, and muscle pain (systemic 1); respiratory symptoms including cough, difficulty breathing, and thoracic pain; and a further, systemic (systemic 2) combination of symptoms including abdominal pain, anosmia, headache, fatigue. Runny nose and sore throat were not related to other symptoms and were not part of any combination (i.e., no edge with any other symptom). Some combinations of symptoms were not related (e.g., systemic 1 and respiratory symptoms), meaning that these symptoms were not likely to occur together. Of note, anosmia did not occur simultaneously with other predictive symptoms or respiratory symptoms.

## Discussion

This cohort study analyzed data from the general population presenting to one of the major test centers in Switzerland during the first COVID-19 wave to establish early predictive clinical symptoms for a positive and negative SARS-CoV-2 test. Based on 712 (21.9%) positive cases among a population of 3,248 patients, a multivariate analysis found that the best predictors for a positive SARS-CoV-2 test results were anosmia, fever, muscle pain, and cough. These results suggest that the clinical definition for mild-to-moderate COVID-19 in the early stage should include these symptoms. By contrast, difficulty breathing, sore throat, abdominal pain, thoracic pain, and runny nose were all associated with negative test results.

Our findings regarding symptoms predictive of a positive test are consistent with those identified by other studies focused on specific populations (16, 17), as well as a recent (16 December, 2020) WHO statement that patients with anosmia may represent a probable case of SARS-CoV-2 infection. Of note, our results regarding symptoms predictive of a negative test result were not consistent with other studies on specific populations, such as US veterans or healthcare workers, who found that diarrhea and difficulty breathing were predictive for a positive test (16, 17). One reason could be that as our patient cohort tended to present for testing early in the course of the disease, difficulty in breathing and diarrhea tended to appear later, as reported by the authors of these studies. Another reason for this inconsistency may be that breathing difficulties in our study were self-reported. The recent WHO definition also suggests that headache should be considered as a suspicion of SARS-CoV2 infection. This was not confirmed in our multivariate analysis.

Our findings should feed into ongoing discussions regarding future public health approaches to containing further COVID-19 outbreaks. Viral loads tend to be elevated at the beginning of COVID-19 infection. It is therefore crucial to define positive and negative clinical predictors during the early stage of the disease to facilitate early diagnosis, isolation, contact tracing, and consequently, interruption of transmission. As our understanding of this novel disease evolves, establishing a clear case definition will help patients, healthcare providers and public health authorities to make informed decisions regarding early diagnosis and isolation measures with a positive impact on the epidemiological spread.

Beyond the impact on public health and onward transmission, a clear case definition will also provide a powerful decision tool for resource-limited settings or situations. During the second wave in autumn 2020, many European settings, including the Geneva region, had to face unprecedented pressure. The high and rising caseloads of positive and suspected cases generated a bottleneck in the provision of molecular testing due to the large patient volume and limited laboratory capacity, resulting in a delay in testing of several days. Similar to the first wave, key decisions had to be made regarding limiting the provision of tests for a given period to those considered most at risk in order to preserve precious resources and prevent test centers and laboratories from being completely overwhelmed. The availability of data such as presented here is vital to make evidence-based decisions regarding such restrictive measures, while ensuring a robust and strong public health response. It goes without saying that large-scale testing remains an important public health intervention to control the epidemic, but only when resources - both human and laboratory-based – allow for this.

In addition to identifying predictive and non-predictive symptoms, this study also provides critical information on the clinical forms of SARS-CoV-2 infection. The network analysis identified three distinct combinations of symptoms that were not likely to occur together and confirmed that COVID-19 can have very different clinical presentations. Each combination of symptoms has one or more predictive symptoms for a positive test. For example, the network of “systemic 1” symptoms, which corresponds to a flu-like syndrome (muscle pain, chills, and fever), was not associated with respiratory symptoms. Anosmia, which seems specific and the strongest predictor for test positivity, was associated with non-specific symptoms such as digestive symptoms, fatigue, and headache. Results related to cough should be carefully considered because it was not positively related to other symptoms of the same combination but was rather inversely related to other symptoms such as anosmia. Indeed, the presence of negative edges may increase the probably of being in a combination of symptoms (here “respiratory”) and weaken the influence of positive edges (31).

This study analyzed a large cohort of outpatients in one of the largest networks of dedicated test centers in Switzerland, a country hard hit by COVID-19. Detailed questionnaires allowed to collect data on a large number of symptoms and evaluate a large range of clinical features, whereas, previous studies mostly focused on positive cases or hospitalized patients. Hence, this cohort provided an opportunity to establish predictive early clinical symptoms for both positive and negative tests in outpatients who were representative of the general population. It provided also an original analysis of how symptoms are combined.

Our study has some limitations. First, test centers used different versions of the questionnaire over time as these were updated as new data on clinical symptoms became available. Although, symptoms not specifically listed in the earlier versions of the questionnaires could be reported as free text, this may have resulted in the underreporting of some symptoms in the early phase of the study. However, we do not believe that this had a major impact on results. We were able to compute robust statistics in the multivariate regression for all symptoms of interest and our sensitivity analysis (limited to patients who completed the last versions of the questionnaire) showed similar results to the main analysis. Second, the results may suffer from severity bias, with some RT-PCR results showing false negatives, although, it should be noted that we used the current gold standard diagnostic test. Unfortunately, we did not perform any follow-up of patients to confirm the RT-PCR test results by serology testing, but this could be a good focus for future studies. Some symptoms appeared as being non-specific, such as difficulty in breathing, sore throat, abdominal symptoms, thoracic pain, and runny nose. We interpreted “difficulty in breathing” as a subjective symptom linked to anxiety when assessed as a self-reported symptom. Concerning “thoracic pain,” we made a clinical choice to use a generic term permitting to include most types of pain of the thoracic region, including cardiac, respiratory, digestive, and back pain. Regarding the physical examination findings and treatment, we observed a substantial amount of missing data and we decided to exclude these elements from the analysis Therefore, some severe cases may have been missed as we referred these immediately to the emergency department. Third, the interpretation and generalizability of the results are limited by the sample size and geographical range (one single canton). It would be useful to perform similar analyses of symptoms and how they are combined in other settings by using data from subsequent outbreaks to confirm the proposed case definition across regions and time. The same analysis could be done with SARS-CoV-2 variants. Finally, the analyses relied on self-reported symptoms, which may have resulted in some degree of bias. For example, it is likely that anxiety may have acted as a confounder for self-reported breathing difficulty, or that some patients reported non-existing symptoms or a more severe level of symptoms in order to gain access to a test.

## Conclusions

This study identified four early clinical signs associated with a positive SRARS-CoV-2 RT-PCR result in an outpatient setting as well as three combinations of symptoms. The results of this study add crucial information concerning the mild-to-moderate stage of COVID-19 by using the innovative network analysis and including a large cohort of outpatients. In the current context of recurrent epidemic waves, this study contributes important data to inform public health approaches, including a rapid identification of outpatients infected with SARS-CoV-2, as well as raising e awareness of early symptoms that are predictive or non-predictive of a positive RT-PCR.

## Data Availability Statement

The data analyzed in this study is subject to the following licenses/restrictions: on demand we could add the anonymised data. Requests to access these datasets should be directed to herve.spechbach@hcuge.ch.

## Ethics Statement

The studies involving human participants were reviewed and approved by The study was reviewed and approved by the Geneva regional ethics committee. Written informed consent for participation was not required for this study in accordance with the national legislation and the institutional requirements.

## Author Contributions

The study was conceived by HS, FJ, JS, and SB and supervised by FJ, JS, and SB. Data were collected, analyzed, and interpreted by HS, FJ, JS and SB with statistical analyses performed by SB. HS drafted the first version of the manuscript, FJ, VP, LK, MS, AC, FC, IG, JS, and SB provided critical revision of the manuscript. All authors contributed to the redrafting and finalization of the manuscript.

## Conflict of Interest

The authors declare that the research was conducted in the absence of any commercial or financial relationships that could be construed as a potential conflict of interest.
