# Supplementary figures and images for: Network Analysis of Outpatients to Identify Predictive Symptoms and Combinations of Symptoms Associated With Positive/Negative SARS-CoV-2 Nasopharyngeal Swabs
Source: Front Med (Lausanne). 2021 Jul 20;8:685124. doi: 10.3389/fmed.2021.685124 (PMC8329357; doi:10.3389/fmed.2021.685124)

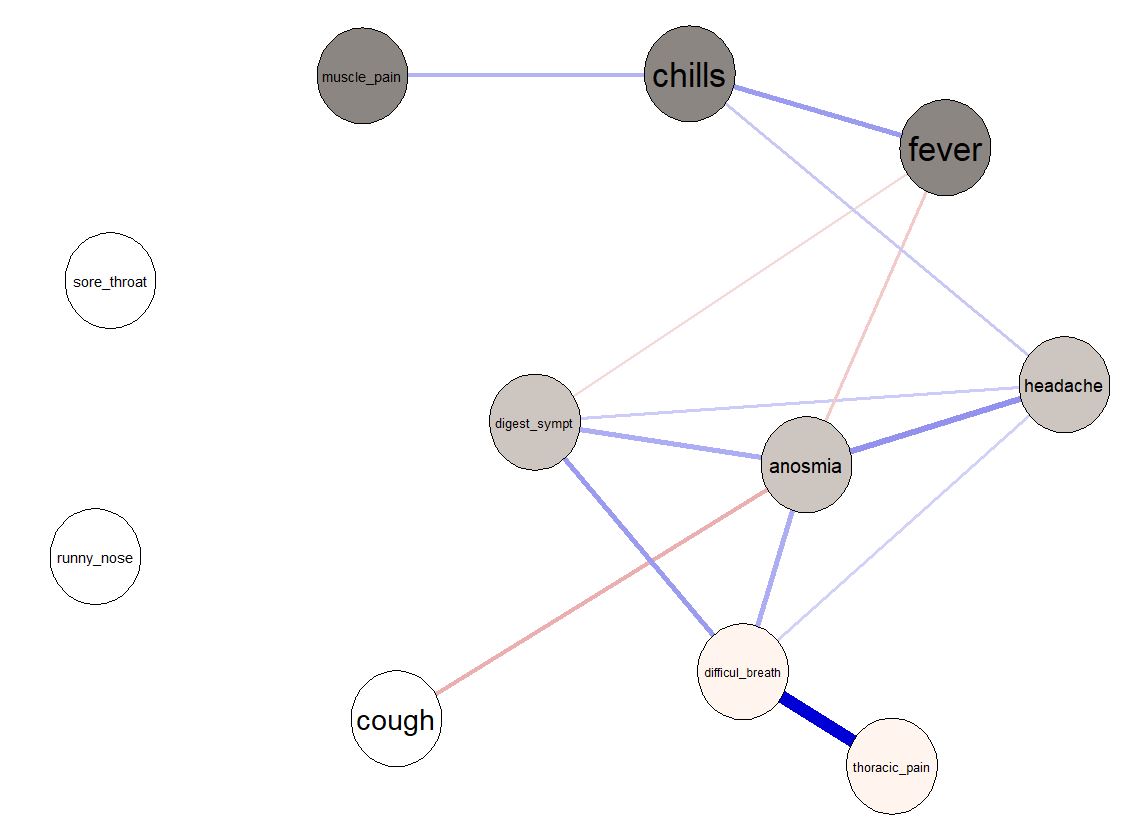

Supplement: Supplementary file 1 [file Image_1.JPEG]
