# Supplementary material for: Network Analysis of Outpatients to Identify Predictive Symptoms and Combinations of Symptoms Associated With Positive/Negative SARS-CoV-2 Nasopharyngeal Swabs
Source: Front Med (Lausanne). 2021 Jul 20;8:685124. doi: 10.3389/fmed.2021.685124 (PMC8329357; doi:10.3389/fmed.2021.685124)
Supplement: Supplementary file 2 [file Table_1.DOCX]

**S1 Table. Sensitivity analyses**

| **Variables** | **Mixed-effect model (n=3,179)** | | **Multiple logistic regression controlling for age and gender (n=3,179)** | | **Multiple logistic regression controlling for the no. of risk factors (n=3,179)** | | **Multiple logistic regression for the subsample of participants with the last version of the questionnaire (n=1,557)** | |
| --- | --- | --- | --- | --- | --- | --- | --- | --- |
|  | **Odd-ratio** | **p-value** | **Odd-ratio** | **p-value** | **Odd-ratio** | **p-value** | **Odd-ratio** | **p-value** |
| Runny nose | 0.52 (0.34; 0.81) | .004 | 0.77 (0.64; 0.93) | .006 | 0.77 (0.64; 0.92) | .005 | 0.96 (0.71; 1.28) | .760 |
| Sore throat | 0.34 (0.22; 0.55) | <.001 | 0.63 (0.53; 0.77) | <.001 | 0.63 (0.52; 0.76) | <.001 | 0.58 (0.42; 0.79) | .001 |
| Muscle pain | 3.30 (2.06; 5.27) | <.001 | 1.73 (1.43; 2.10) | <.001 | 1.72 (1.42; 2.08) | <.001 | 1.31 (0.95; 1.80) | .096 |
| Chills | 0.97 (0.61; 1.53) | .878 | 0.98 (0.81; 1.20) | .870 | 0.99 (0.81; 1.20) | .878 | 0.91 (0.65; 1.27) | .579 |
| Cough | 2.70 (1.67; 4.37) | <.001 | 1.55 (1.26; 1.90) | <.001 | 1.54 (1.26; 1.89) | <.001 | 1.50 (1.11; 2.06) | .011 |
| Fever | 5.13 (3.13; 8.41) | <.001 | 1.97 (1.63; 2.38) | <.001 | 1.94 (1.61; 2.35) | <.001 | 1.51 (1.10; 2.06) | .009 |
| Headache | 1.32 (0.84; 2.08) | .223 | 1.16 (0.95; 1.41) | .149 | 1.11 (0.91; 1.35) | .306 | 1.04 (0.76; 1.42) | .810 |
| Anosmia | 71.82 (31.10; 165.85) | <.001 | 5.96 (4.70; 7.56) | <.001 | 5.82 (4.60; 7.36) | <.001 | 7.83 (5.72; 10.73) | <.001 |
| Abdominal symptoms | 0.44 (0.25; 0.77) | .004 | 0.70 (0.55; 0.89) | .003 | 0.69 (0.54; 0.88) | .002 | 0.63 (0.46; 0.87) | .005 |
| Fatigue | 0.66 (0.40; 1.09) | .104 | 0.83 (0.66; 1.03) | .086 | 0.83 (0.67; 1.03) | .096 | 1.24 (0.91; 1.71) | .178 |
| Difficulty breathing | 0.25 (0.13; 0.46) | <.001 | 0.53 (0.41; 0.69) | <.001 | 0.53 (0.41; 0.69) | <.001 | 0.59 (0.43; 0.82) | .002 |
| Thoracic pain | 0.51 (0.27; 0.96) | .036 | 0.70 (0.53; 0.93) | .014 | 0.70 (0.53; 0.92) | .011 | 0.78 (0.55; 1.12) | .176 |
| Gender (ref. female) | - | - | 0.74 (0.62; 0.89) | .002 | - | - | - | - |
| Age (continuous) | - | - | 1.00 (1.00; 1.01) | .198 | - | - | - | - |
| No. of risk factors | - | - | - | - | 0.88 (0.77; 1.00) | .058 | - | - |
